# Marine-Derived Compounds with Potential Use as Cosmeceuticals and Nutricosmetics

**DOI:** 10.3390/molecules25112536

**Published:** 2020-05-29

**Authors:** Ana Alves, Emília Sousa, Anake Kijjoa, Madalena Pinto

**Affiliations:** 1 Laboratório de Química Orgânica e Farmacêutica, Departamento de Ciências Químicas, Faculdade de Farmácia, Universidade do Porto, Rua de Jorge Viterbo Ferreira 228, 4050-313 Porto, Portugal; anajoao93@hotmail.com (A.A.); esousa@ff.up.pt (E.S.); 2 Centro Interdisciplinar de Investigação Marinha e Ambiental (CIIMAR), Universidade do Porto, Terminal de Cruzeiros do Porto de Leixões, Avenida General Norton de Matos s/n, 4450-208 Matosinhos, Portugal; 3 ICBAS-Instituto de Ciências Biomédicas Abel Salazar, Universidade do Porto, Rua de Jorge Viterbo Ferreira 228, 4050-313 Porto, Portugal

**Keywords:** cosmeceuticals, nutricosmetics, marine-derived compounds, anti-tyrosinase, antiaging, anti-wrinkle, UV protection

## Abstract

The cosmetic industry is among the fastest growing industries in the last decade. As the beauty concepts have been revolutionized, many terms have been coined to accompany the innovation of this industry, since the beauty products are not just confined to those that are applied to protect and enhance the appearance of the human body. Consequently, the terms such as cosmeceuticals and nutricosmetics have emerged to give a notion of the health benefits of the products that create the beauty from inside to outside. In the past years, natural products-based cosmeceuticals have gained a huge amount of attention not only from researchers but also from the public due to the general belief that they are harmless. Notably, in recent years, the demand for cosmeceuticals from the marine resources has been exponentially on the rise due to their unique chemical and biological properties that are not found in terrestrial resources. Therefore, the present review addresses the importance of marine-derived compounds, stressing new chemical entities with cosmeceutical potential from the marine natural resources and their mechanisms of action by which these compounds exert on the body functions as well as their related health benefits. Marine environments are the most important reservoir of biodiversity that provide biologically active substances whose potential is still to be discovered for application as pharmaceuticals, nutraceuticals, and cosmeceuticals. Marine organisms are not only an important renewable source of valuable bulk compounds used in cosmetic industry such as agar and carrageenan, which are used as gelling and thickening agents to increase the viscosity of cosmetic formulations, but also of small molecules such as ectoine (to promote skin hydration), trichodin A (to prevent product alteration caused by microbial contamination), and mytiloxanthin (as a coloring agent). Marine-derived molecules can also function as active ingredients, being the main compounds that determine the function of cosmeceuticals such as anti-tyrosinase (kojic acid), antiacne (sargafuran), whitening (chrysophanol), UV protection (scytonemin, mycosporine-like amino acids (MAAs)), antioxidants, and anti-wrinkle (astaxanthin and PUFAs).

## 1. Introduction

The European Commission (EC) regulation No.1223/2009 defines cosmetics as “products intended to be applied to the external parts of the human body such as epidermis, hair, nails, lips and external genital organs, or teeth and the mucous membranes of the oral cavity with the exclusive or principal objective to clean, perfume, protect or change their appearance or keep them in good conditions” [1]. Although, cosmetics are not intended to affect the structure and function of the body, there are many requirements for cosmetics, including safety, lack of side effects, and their ability to show positive effects on well-being [2]. As the market of cosmetics is highly dynamic and new products are being constantly launched in an extremely fast rate, new concepts have also been continuously emerged, and new terms have been coined. Thus, the term “cosmeceuticals”, which derives from a combination of “cosmetics” and “pharmaceuticals”, and was popularized by Kilgman [3], refers to cosmetic products with drug-like benefits. Although the Federal Food, Drug and Cosmetic Act (FD&C Act) does not recognize this term, it is widely used in the cosmetic industry [4]. In turn, the most recent concept, representing the latest trend in the beauty industry is “nutricosmetics”, which is emerged from a combination of “cosmeceuticals” and “nutraceuticals”, and these are destined for the oral supplementation of nutrients formulated and marketed specifically for beauty purposes [4]. Nutricosmetics are characterized as natural health products with a capacity to improve the function and appearance of the skin, hair, and nails when ingested. It is believed that these compounds exert their beautification effects and/or personal hygiene within the body. Thus, nutricosmetics are becoming a strong trend, since consumers today have a great awareness of the foods and food supplements, tending to acquire preferentially products from natural origin that can restore and improve health and beauty without posing any prejudicial effects [4].

Cosmeceuticals comprise active ingredients such as vitamins, minerals, phytochemicals, enzymes, which exist in various types of formulations such as creams, lotions, and ointments [5]. These natural bioactive substances can derive from diverse sources such as terrestrial plants, microorganisms, and marine organisms. These substances can have a myriad of functional roles including those with beneficial effects on human health [5], which can promote healthy skin, hair, and nails at cellular levels [6]. Although plant-derived ingredients are still very popular and widely used as cosmeceuticals, they also have some limitations because plants generally grow too slowly and their chemical composition varies from season to season and from region to region. On the contrary, marine flora and fauna not only produce chemically unique biomolecules not found in terrestrial resources but also can be grown rapidly in large quantities and cost effective by modern aquaculture techniques [7].

## 2. Biological Targets and Mechanisms of Action of Cosmeceuticals

Currently, there is a great demand of cosmeceuticals that function as skin depigmentation, UV filters, anti-inflammatory, anti-wrinkle, antiaging, skin hydrating, antiacne, as well as antioxidant and cytoprotective agents [8]. Therefore, this section will briefly discuss the biological activities and underlying mechanisms of action of some major cosmeceuticals as well as biochemical pathways and targets involved in these processes.

### 2.1. Antimelanogenic Activity

**Figure 1:**
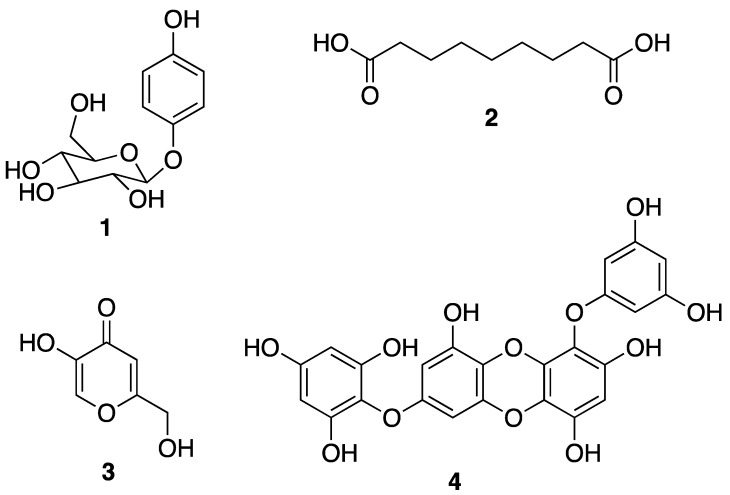
Structures of arbutin (**1**), azelaic acid (**2**), kojic acid (**3**), and 7-phloroeckol (**4**).

The demand for skincare products is motivated by the intention to brighten and lighten the skin tone as well as to eliminate local hyperpigmentation [9]. Skin whitening involves the use of natural or synthetic substances that cause a decrease in pigmentation by reducing the melanin concentration in the skin. This practice may be driven by dermatological needs such as skin hyperpigmentation caused by autoimmune conditions, exposure to UV radiation, genetic factors, and hormonal changes that can induce an overproduction of melanin in the skin [10]. The depigmentation process can involve one or more steps in the melanogenic pathway, such as melanosome transfer or post-transfer pigment processing, and degradation. Therefore, melanin biosynthesis can be prevented by avoiding UV exposure, the inhibition of the tyrosinase enzyme, melanocytes metabolism and proliferation, or removing melanin itself [11]. Skin whitening can be achieved by several mechanisms, such as the inhibition of microphthalmia-associated transcription factor, downregulation of melanocortin 1 receptor activity, interference with melanosome maturation and transfer, melanocyte loss, and inhibition of the tyrosinase enzyme [12]. Several depigmenting agents modulate skin pigmentation by influencing the transcription and activity of tyrosinase-related melanogenic enzymes, tyrosinase-related protein-1 (TYRP-1), tyrosinase-related protein-2 (TYRP-2), or peroxidase [13]. Tyrosinase inhibition has become the most common and increasingly popular in skin whitening cosmetic products. Until now, the use of synthetic tyrosinase inhibitors is rather limited owing to their toxicity, low stability, poor skin penetration, and low activity [14]. Traditionally, compounds from plants such as a hydroquinone glycoside arbutin (**1**) and azelaic acid (**2**), as well as from fungi such as kojic acid (**3**) (Figure 1) [10], have been widely used as skin whiteners in cosmetics. However, in recent years, the research has been focused on compounds from marine organisms, especially phlorotannins such as 7-phloroeckol (**4**) from brown algae (Figure 1), since it is generally believed that these compounds are safer than the conventional skin whiteners. The safety question originated from the idea that the active principles are not isolated, but instead exist in a complex and stable chemical clusters that prevent their negative effects on the site of application [15].

#### Anti-Tyrosinase Activity

Skin pigmentation is the most important photoprotective factor, since melanin does not only function as a broadband UV absorbent but also possesses antioxidant and radical scavenging properties [16]. In addition, melanin also plays an important role in camouflage, heat regulation, and cosmetic interaction. Pigmentation is highly heritable and regulated by genetic, environmental, and endocrine factors that modulate the amount, type, and distribution of melanin in the skin, hair, and eyes. As the skin is the largest organ of the body that is always subject to internal and external conditions, it often responses to these factors by modifying the constitutive pigmentation pattern [17]. Consistently, overproduction or a lack of melanin pigment is not just an aesthetic problem since minor changes in the physiological status of the human body or exposure to harmful external factors can affect pigmentation patterns either in transitory (such as in pregnancy) or permanent (e.g., age spots) manners [17]. For this reason, there is also a great demand for whitening cosmetics for the treatment of lentigo, pregnancy mask, or even hyperpigmentation caused by medicine poisoning.

Melanin is produced by sequential enzymatic processes in melanosomes, an organelle residing in melanocytes, and then transferred to nearby keratinocytes for photoprotection [18,19]. Tyrosinase is a multifunctional, membrane glycosylated and copper-containing oxidase enzyme that intervenes in the early stages of melanogenesis by the hydroxylation of tyrosine to 3,4-dihydroxyphenylalanine (DOPA) and subsequently oxidizes DOPA to dopaquinone [20]. Since tyrosinase is the rate-limiting enzyme, it is critical for melanin synthesis and controls the pigmentation in the skin. Thus, the inhibition of this biological target is currently the most common approach for the development of skin whitening agents for cosmetics [18].

Despite a large number of compounds exhibiting the in vitro tyrosinase inhibitory activity, only a few were effective in clinical trials [21,22]. Thus, understanding the mechanisms by which different factors and compounds induce melanogenesis is fundamental to design and develop products with particular purposes such as pigmentary diseases therapy and tanning products to reduce skin cancer risk, among others [17]. Furthermore, tyrosinase was also reported to catalyze the formation of dopamine quinone in human substantia nigra, which is a substance that may be involved in a dopamine neurotoxicity and various neurodegenerative diseases such as Parkinson’s disease. Consistently, tyrosinase might also be a potential target for drug development for the treatment of Parkinson’s disease [23]. The discovery of novel tyrosinase inhibitors with an ability to regulate melanogenesis is of special interest, as the excessive production of melanin leads to hyperpigmentation of the skin in the form of freckles, the so-called “age spots” and melanoma. Although several tyrosinase inhibitors belonging to different chemical classes have been discovered from marine resources as skin whitening agents or for the treatment of pigmentation disturbances, some of them have negative effects on human health [24].

Another important aspect is that although the inhibitory strength of tyrosinase inhibitors is normally expressed as their values of half inhibitory concentration (IC_50_), it is not possible to directly compare the inhibitory activity of different compounds from their IC_50_ values reported in the literature, since the experimental conditions such as substrate concentrations, incubation time, and the batches of commercial tyrosinase enzymes used varied among different assays. To avoid the discrepancy, most studies conducted to evaluate new tyrosinase inhibitors use a standard tyrosinase inhibitor such as kojic acid (**3**) (Figure 1) as a positive control [20]. Kojic acid (**3**), a fungal metabolite currently used as a skin whitening agent in cosmetic and also as a food additive to prevent enzymatic browning, is the most intensively studied tyrosinase inhibitor [25].

As tyrosinase inhibitors are not only important depigmentation agents in cosmetics but also clinically useful for the treatment of some dermatological diseases associated with melanin hyperpigmentation [26], it is important to correctly define the term “tyrosinase inhibitor”. Generally, the designation of “tyrosinase inhibitor” is not always very clear, because some authors use the same terminology to refer to inhibitors of melanogenesis whose action mainly involves interference in melanin formation but without any direct effect on the tyrosinase enzyme. Thus, only specific inactivators and/or specific inhibitors of tyrosinase, which bind directly to the enzyme and inhibit its activity, are considered “true inhibitors”. These “true inhibitors” of tyrosinase are then divided into two categories: (1) specific tyrosinase inhibitors that bind reversibly to the enzyme, thus reducing its catalytic capacity [20], and (2) specific tyrosinase inactivators, also known as irreversible inhibitors or “suicide substrates”, which form a covalent bond to tyrosinase, thus altering its active site and inactivating the enzyme irreversibly during the catalytic process (e.g., L-DOPA and catechol). Most importantly, these compounds are generally specific for tyrosinase and do not inactivate other proteins [27,28]. Tyrosinase inhibitors can be also categorized, based either on their chemical structures or inhibitory mechanisms, into five major classes: (1) polyphenols, (2) benzaldehyde and benzoate derivatives, (3) long-chain lipids and steroids, (4) other natural or synthetic inhibitors, and (5) irreversible inactivators. Polyphenols represent the most diverse and largest group of tyrosinase inhibitors, flavonoids being the major representative of this group [29]. In addition to flavonoids, several stilbenes and coumarin derivatives are found to have anti-tyrosinase activity [30].

### 2.2. Antiaging Activity

The term “skin aging” refers to the degradation of the dermis, including thinning, dryness, laxity, fragility, enlarged pores, fine lines and wrinkles, vasculature prominences, increase in transparency, and loss of elasticity [31]. The aging process reduces skin thickness, elasticity, and curling of elastic fibers in the skin, which gives rise to wrinkles [32]. Intrinsic aging is generally determined by genetic factors; however, extrinsic factors such as exposure to sunlight, pollution or nicotine, repetitive muscle movements such as squinting or frowning, and lifestyle such as diet, sleeping position, and overall health also contribute to the aging process [31]. Aging is also influenced by a decrease in collagen gene expression, low fibroblast activity, and fibroblast regeneration as well as shrinking of the lamellar barrier, which results in the inability of the skin to retain moisture. Although the mechanisms underlying skin aging are not completely elucidated, the cosmetic industry continues to offer an enormous variety of antiaging products, most of which is claimed to stimulate collagen and glycosaminoglycan (GAG) synthesis by fibroblasts in the epidermis, thus increasing the firmness and flexibility of the corneal layer of the skin [33].

**Figure 2:**
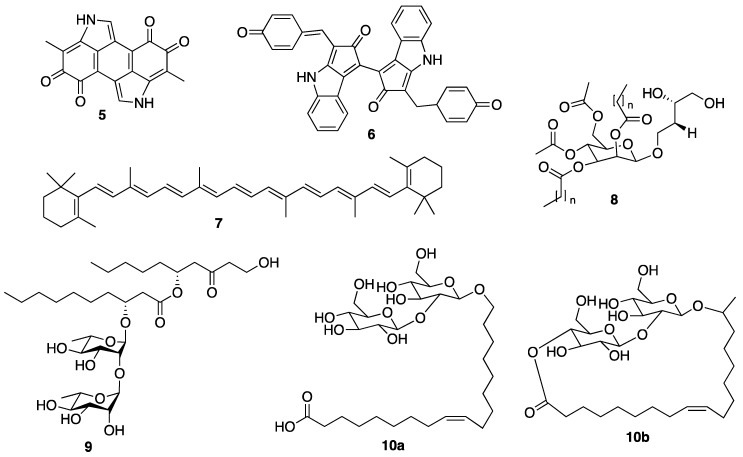
The structures of melanin (**5**), scytonemin (**6**), β-carotene (**7**), mannosylerythritol (**8**), rhamnolipid (**9**), and sophorolipids (free acid type (**10a**) and lactone type (**10b**)).

Human skin is the anatomical barrier for pathogens and physical damages, acting as a partition between internal and external environments [34,35]. The skin protects our body from external aggressors, especially the sun, which involves a series of mechanisms that minimize the damages when exposed to UV radiation. These mechanisms can be controlled by certain organic and inorganic compounds, e.g., melanin (**5**) (Figure 2) [34,35]. Different organisms produce different chemicals to protect themselves from the deleterious effects of UV radiation. For example, while animals (including humans) use melanin (**5**) to protect themselves from UV radiation, higher plants produce secondary metabolites such as flavonoids, and microorganisms that live in the marine environments with high volume of sunlight produce compounds such as scytonemin (**6**) (Figure 2), mycosporine-like amino acids (MAAs), and several other UV-absorbing substances of unknown chemical structure for the same purpose [36,37]. Carotenoids, another class of UV filters produced by many species of microalgae, are also major active compounds among the top ingredients with antiaging properties [38], among which β-carotene (**7**) (Figure 2) is one of the most effective compounds to prevent reactive oxygen species (ROS) formation, thus avoiding cellular damage and the aging process [39].

#### 2.2.1. Antiphotoaging Activity

Chronic exposure to UV radiation can cause dermatoheliosis or photoaging [40]. Exposure to UV irradiation, both UVA (400 nm < λ< 320 nm) and UVB (320 nm < λ < 290 nm), can lead to alterations in the composition of the dermal extracellular matrix (ECM), resulting in wrinkles, laxity, coarseness, mottled pigmentation, and histological changes including epidermal thickness and connective tissue alteration or even skin cancer (melanoma), which are typically mediated by ROS [41,42,43]. Continuous exposure to UV radiation leads to numerous complications that are correlated with various pathological consequences of the skin damage. For example, sunburn occurs when exposure to UV radiation exceeds the protective capacity of an individual’s melanin [43,44,45,46]. Although short-term solar exposure can be beneficial on mood and vitamin D synthesis, it can also cause an immediate skin burn, detrimental skin thickening, actinic erythema, and excessive tanning. On the other hand, the long-term effects are all negative, including photo-induced skin aging and photo-carcinogenesis caused by UV radiation-induced immunosuppression. The severity of these long-term effects requires the use of an appropriate protection during UV radiation exposure [47]. Although UVB affects mainly the epidermis and UVA intervenes directly in the dermal compartment, both are the major factors responsible for the photoaging of human skin, damaging dermal fibroblasts, through the induction of cytokines, matrix metalloproteinases (MMPs), and mitochondrial DNA mutations [48,49]. Radiation-induced oxidation may cause photoaging by the reduction of antioxidant enzymes and the antioxidant defense mechanism, which may result in significant oxidative damage, immunomodulation, the activation of melanogenesis, and ultimately carcinogenesis [50]. To avoid the deleterious effects caused by UV exposure, sunscreen products that commonly contain organic and/or inorganic filters are used [51,52,53]. However, a number of naturally occurring photoprotective compounds such as scytonemin (**6**, from cyanobacteria), mycosporines (from fungi and cyanobacteria), MAAs (from cyanobacteria, microalgae, macroalgae, yeasts, fungi, sponges, corals, and animals), flavonoids (from higher plants), melanins (in humans and other animals and even some bacteria), and several other UV-absorbing substances of unknown chemical structures from different organisms have been explored to develop novel UV filters for sunscreen products to prevent the photodamage [54,55,56].

A variety of photosynthetic organisms have been investigated as sources of photoprotective compounds. These include mycosporines, MAAs, and several other UV filters [42,56,57]. MAAs belong to a family of secondary metabolites produced by a variety of organisms, especially those inhabit ecosystems with a high amount of sunlight such as marine and freshwater environments, for protection against solar radiation [58]. These low molecular weight (usually <400 Da) and colorless compounds are water soluble and share the same chemical scaffold, but they differ in substituents and/or the presence and type of amino acids. Their structures consist of cyclohexenone or cyclohexenimine chromophores linked to a nitrogen substituent of an amino acid or its iminoalcohol by a conjugation [58,59]. MAAs absorb UV radiation ranging from 310 to 362 nm, and dissipate this energy in the form of heat radiation to the surrounding environment [60]. The protection efficiency of MAAs against UV radiation depends also on their location in the cell, i.e., MAAs located in the cytoplasm provide a limited protection against UV radiation while extracellular MAAs are more effective protector [61,62]. On the other hand, scytonemin (**6**) (Figure 2), a stable yellow-brown and lipid-soluble pigment, is located in the extracellular polysaccharide sheath of some cyanobacteria. Scytonemin (**6**) has a maximum absorption at 386 nm, but also absorbs significantly at 252, 278, and 300 nm. Recent studies suggested that scytonemin (**6**) not only has a potential as a UV filter in cosmetics but also as an anticancer drug [63].

#### 2.2.2. Anti-Wrinkle Activity

A number of investigations revealed that MMPs, a family of secreted or transmembrane zinc endopeptidases, are responsible for the inhibition of collagen synthesis in photoaged skin [64]. MMPs are produced by a variety of cells, including fibroblasts, keratinocytes, mast cells, macrophages, and neutrophils, and they are believed to play a major role in wrinkle formation [65,66]. MMPs can be sub-divided in three major functional groups, i.e., interstitial collagenases (which degrade types I, II, and III collagen) [67], stromelysins (which degrade laminin, fibronectin, and proteoglycans) [68], and gelatinases (which degrade type IV and V collagens) [69]. MMPs expression is usually induced by various extracellular stimuli such as growth factors, cytokines, and UV radiation [70,71]. MMP gene expression can be also influenced by ROS through the signal transduction pathway [72]. Moreover, MMPs overexpression is associated with tissue remodeling, repair, and destruction phenomena. For example, MMP-2 and MMP-9 can degrade ECM and influence the formation of wrinkles and skin thickness [73]. The induction of collagenase or MMP-1, leading to collagen type I degradation, can enhance wrinkle formation, and since collagen type I is a major constituent of the connective tissue, it cannot be compensated by a concomitant induction of collagen synthesis [74]. This imbalance is usually increased by the influence of UVA irradiation, resulting in a decreased expression of collagen 1A1 and collagen 1A2, which induces the upregulation of cytokine interleukin (IL)-6 [75]. On the other hand, the transcription factor, activating protein-1 (AP1), which is activated upon UVA stimulation, induces the MMP-1 synthesis and the repression of collagen 1A1 and collagen 1A2 [76]. Accordingly, MMPs are useful markers for skin aging and agents that stimulate collagen synthesis and/or reduce the photo-induced upregulation of MMPs are potentially useful for skincare products [33]. Interestingly, several studies have revealed that nutrient-derived compounds such as chitooligosaccharides, flavonoids, polyphenols, and fatty acids are able to inhibit the activation and expression of MMPs [71,77,78]. Therefore, these compounds could have a strong potential for the development of nutricosmetic products.

All the anti-wrinkle/antiaging cosmetic formulations normally contain moisturizing components to maintain skin hydration, which is essential for skin functions. The external application of lipids that limit water loss, or of compounds with a capacity to form bonds with water molecules are used to mimic the natural hydrating mechanisms of the skin [10]. Traditionally, linoleic acid and γ-linolenic acid are commonly used for the oil/water emulsion to retain the water in the skin in order to restore transepidermal water loss (TEWL) to its normal level [79]. However, recently, some marine microorganisms-derived biosurfactants such as mannosylerythritol (**8**), rhamnolipids (**9**), and sophorolipids (**10a** and **10b**) (Figure 2) are being investigated for their application in the cosmetic industry due to their emulsifying, solubilizing, wetting, foaming, and dispersing properties, which can not only enhance the solubilization of hydrophobic ingredients in the products but also facilitate their delivery through the skin barrier [80]. Moreover, these marine-derived biosurfactants have an advantage over their synthetic counterparts, since they have low irritancy to the skin, which is ideal for the anti-wrinkle formulations [81].

### 2.3. Antioxidant Activity

**Figure 3:**
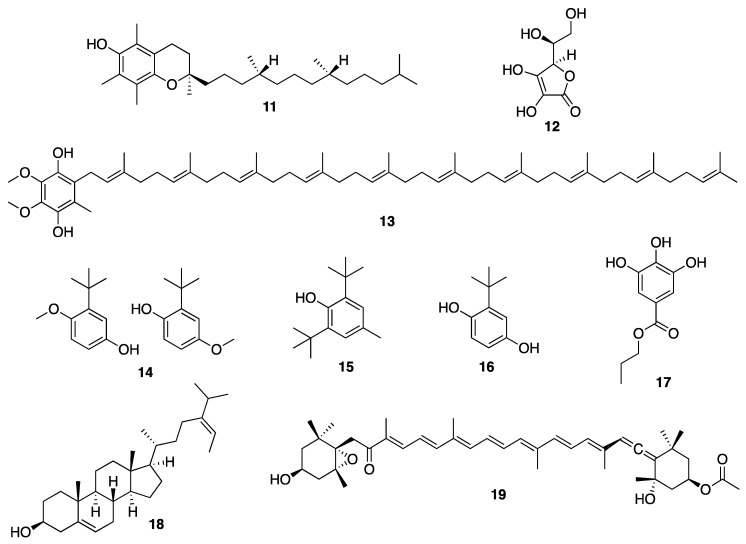
Structures of R-tocopherol (**11**), ascorbic acid (**12**), ubiquinol (**13**), hydroxyanisole (**14**), butylated hydroxytoluene (**15**), tertiary butylhydroquinone (**16**), propyl gallate (**17**), fucosterol (**18**), and fucoxanthin (**19**).

Antioxidants play an important role in cellular protection against aging by preventing UV-induced ROS such as superoxide anion (O_2_^−^), hydroxyl radical HO^.^), and H_2_O_2_ to attack membrane lipids, proteins, and DNA [82,83]. Since the oxidation of membrane lipid is one of the most important factors that decreases the youthful appearance of the skin [84], the prevention of ROS formation is fundamental. Antioxidants provide protection against the pro-oxidative environment to which human skin is exposed, in particular, UV radiation, smoke, and air pollutants [82,83]. Therefore, the consumption of antioxidant-rich food supplements is an important strategy used in the so-called “antioxidant therapy” to maintain health as well as to prevent many diseases. Antioxidants consist of enzymatic and non-enzymatic molecules. Enzymatic antioxidants include superoxide dismutase (SOD), catalase (CAT), glutathione peroxidase (GSH), glutathione reductase (GR), and glutathione transferase (GST), which are present in human plasma and erythrocytes [85,86]. Non-enzymatic antioxidants consist of many classes of small molecules such as β-carotene (**7**) (Figure 2), *R*-tocopherol (TOH) (**11**), ascorbic acid (**12**), and ubiquinol (**13**) (Figure 3), among others [87].

Currently, many synthetic antioxidants such as butylated hydroxyanisole (BHA) (**14**), butylated hydroxytoluene (BHT) (**15**), *tert*-butylhydroquinone (TBHQ) (**16**), and propyl gallate (**17**) (Figure 3) are used as additives to suppress oxidation in food, cosmetics, and drugs. However, the use of these synthetic antioxidants for food or drugs has been restricted, because they may lead to potential problems in human health due to their toxicity and lack of safety [88,89]. On the contrary, since natural antioxidants are considered as safe alternatives, many research efforts have been carried out to discover effective natural antioxidants for the cosmetics industry [90,91,92,93]. Natural antioxidants such as phlorotannins, sulfated polysaccharides, fucosterol (**18**), and fucoxanthin (**19**) (Figure 3), all derived from macroalgae, are believed to be good alternatives for the cosmetics industry [8,94].

Natural pigments such as chlorophylls, carotenoids, and tocopherol derivatives such as vitamin E and isoprenoids are also interesting natural antioxidants that can be obtained from marine resources [90,91,92,93]. The antioxidant and anti-inflammatory properties of carotenoids, which contribute to their photoprotection of the skin through inhibition of UVA-induced ROS toxicity, make them major ingredients in many sunscreen lotions [53]. On the other hand, MAAs can not only protect the skin against UV radiation but also exhibit a high antioxidant activity by scavenging superoxide anion, and therefore prevent lipid peroxidation [95,96,97]. The properties of MAAs as UV filters and ROS scavengers suggest that they could be very useful ingredients for sunscreen products [98]. Another interesting class of natural antioxidants is marine-derived oligosaccharides and peptides. Algae-derived carbohydrates have been suggested to have, besides their thickening and moisturizing properties, antioxidant, anti-melanogenic, and antiaging properties, which are beneficial to skin, therefore representing value-added cosmeceuticals [99,100].

### 2.4. Antiacne Activity

**Figure 4:**
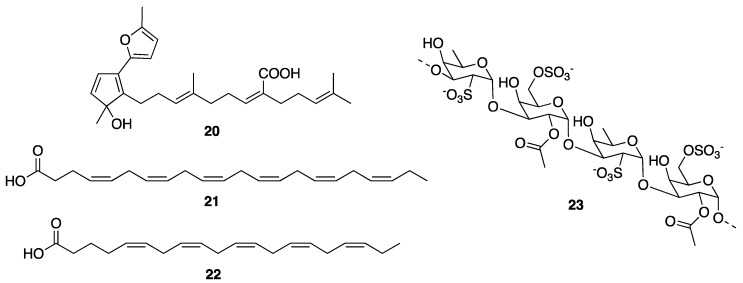
Structures of sargafuran (**20**), docosahexaenoic acid (**21**), eicosapentaenoic acid (**22**), and fucoidan (**23**).

Acne vulgaris, commonly known as acne or pimples, which is a typical condition of adolescence but can also happen in adults, is the most common skin disorder characterized by the inflammation of the sebaceous glands [101]. Acne is caused by multi-factorial events including hormonal, microbiological, and immunological mechanisms such as the androgen-mediated stimulation of sebaceous gland activity, follicular hyperkeratinization, and inflammation. The bacterium *Propionibacterium acnes* is a causative agent of the inflammatory stage and thus initiates the inflamed lesion [102,103,104]. Therefore, *P. acnes* and *Staphylococcus* *epidermidis* are the main targets for the prevention and medical treatment of acne [101,103]. These anaerobic bacteria stimulate a production of pro-inflammatory cytokines and induce the release of ROS whose excessive production results in a destructive phenomenon leading to scarring [105]. They also release lipases to digest a surplus of the skin oil and sebum, which in turn stimulates an intense local inflammation that bursts hair follicles. Therefore, the inhibition of the growth of *P. acnes* has been recognized as a strategic method for treatment of acne in the cosmetics industry. In the search for new antibacterial compounds against *P. acnes* from marine bioresources to develop new natural cosmetic products to prevent acne, sargafuran (**20**) (Figure 4), isolated from the extract of a marine brown alga *Sargassum macrocarpum*, was found to exhibit potent antiacne activity against *P. acnes* with a minimum inhibitory concentration (MIC) value of 15 µg/mL [106]. In an effort to find mari (ne-derived compounds to treat acne vulgaris, Choi et al. have evaluated the antibacterial activity of various species of macroalgae, commonly found around the coast of South Korea; however, only *Ecklonia kurome*, *E. cava,* and *Ishige sinicola* exhibited strong growth inhibitory activity against *P. acnes* as well as an anti-inflammatory activity [107]. Therefore, compounds produced by these three algal species could be promising agents for the development of cosmetic products to combat acne vulgaris.

### 2.5. Wound Healing and Anti-Inflammatory Activities

Wound healing is a complex and tightly regulated process of recovering the forms and anatomical functions of injured tissues, which consists of three overlapping phases [108,109]. The initial inflammatory phase, characterized by platelet activation and the release of growth factors and cytokines, is followed by the proliferative phase, where growth factors are secreted and cell proliferation is enhanced, and finally the last phase consisting of the remodeling, in which collagen production and organization take place, which leads to the mature scar [110]. Acute or normal wound healing proceeds through the orderly overlapping processes, allowing for repair of the skin function and integrity in a coordinated manner in healthy individuals, in a period of 7 to 10 days [111]. The smooth progression of all these events will lead to a normal completion of wound healing and restore the disrupted functions of the skin [112,113]. However, any changes that interrupt the healing process can aggravate tissue damage and delay the repair process, contributing to chronic wound healing. Various factors, such as infections, underlying diseases (e.g., diabetes or cardiovascular disease), medications (e.g., steroids) and old age can impair the wound-healing process [109,111]. On the other hand, inflammation is a vital event of the body’s immune response that involves the interaction of a complex cascade of various cells, including leukocytes, blood cells, fibroblasts, and epithelial cells [108]. There are two types of signals in the inflammatory process: those that start and maintain the inflammation, and the others that stop the process, and the asymmetry of these signals can cause cell and tissue damage. Thus, the deregulation of the whole process can lead to chronic inflammation and even to death in some cases [114]. Inflammatory skin diseases are very common dermatological problems that exist in a variety of forms, i.e., from occasional rashes accompanied by skin itching and redness to more chronic conditions such as atopic dermatitis, rosacea, seborrheic dermatitis, and psoriasis [115]. Cutaneous inflammation has been linked to many diseases, including cancer and discoid lupus erythematosus (DLE) as well as visible anticipated skin aging. However, visible skin aging can be reduced and prevented by a daily use of antioxidants or anti-inflammatory cosmeceuticals, coupled with a diet rich in anti-inflammatory and antioxidant supplementation [116]. Microbiological and immunological factors and toxic agents can initiate the inflammatory response by activating a variety of humoral and cellular mediators such as prostaglandins (PGs), leukotrienes (LTs), NO, tumor necrosis factor alpha (TNF-α), and cytokines of the interleukin (IL) families [117]. Dermal and epidermal cells constitutively produce various cytokines and eicosanoids that play a crucial role in the maintenance of homeostasis and regulation of skin inflammation, and whose levels are regulated by physiological and pathophysiological events [118,119]. Arachidonic acid (AA), a precursor of the pro-inflammatory eicosanoids, is released from membrane phospholipids in the course of inflammatory activation and then metabolized to PGs and LTs [118,119]. Various strategies have been investigated to control the excessive production of lipid mediators on different levels of biochemical pathways, such as the inhibition of phospholipase A2 (PLA2), triggering of enzyme for AA release, blockage of cyclooxygenase (COX) and lipoxygenase (LOX) pathways, and the development of receptor antagonists against platelet-activating factor (PAF) and LTs [118,119]. Most conventional treatments for dermal wounds such as nonsteroidal anti-inflammatory drugs (NSAIDs), immunomodulatory drugs, and topical corticosteroids aim to reduce inflammation [120]. However, this treatment can have a negative impact on wound healing, including adverse effects such as atrophy, osteoporosis, obesity, and glaucoma [121]. Although it is pivotal to search for new anti-inflammatory agents with less adverse effects, the endeavor is quite challenging due to the complexity of the inflammatory process and its role in the host defense. However, recent progress to unravel the mechanisms involved in inflammation has allowed the identification of new targets [122].

Considering the marine resources, none can compare with sea cucumbers in terms of wound-healing properties [123,124,125]. Sea cucumbers, especially *Stichopus hermanni*, commonly known as “gamat emas”, have long been recognized in the folk medicine for the treatment of a myriad of diseases, including wound healing [126]. In vivo studies, using various types of animal models, demonstrated that wounds treated with sea cucumber extracts were better and more rapidly healed when compared to those without treatment. The topical application of extracts of various species of sea cucumbers to wounds induced in animals was found to accelerate the wound contraction rate, which is a fundamental process in a wound-healing phase [127]. Moreover, treating a burn wound with a *S. hermanii*-based hydrogel wound dressing resulted in a significant wound contraction rates at day 21 and 28 post-burn wound. On the contrary, no significant differences were detected at day 7 and 14 [128]. This effect might be due to the cross-linked “gamat hydrogel (*S. hermanii*) dressing” that confers its capacity to retain the active ingredients and delays their delivery on the wounded skin, thus acting at a later stage of the wound-healing phase. The advantage of this hydrogel dressing is that biologically active compounds are immobilized for a longer period in the hydrogel matrices, thus creating a sustained and controlled release system that could significantly enhance the activity of the incorporated sea cucumber extract during tissue repair and effectively interact with the wounds and facilitate a healing process at a later stage [128]. Another sea cucumber species, *S. choronotus,* was also found to act at the initial phase of wound healing [125]. Interestingly, its aqueous extract showed antioxidant activity approximately 80% higher than its organic counterpart [129]. Since the presence of excessive free radicals is associated with impaired wound healing, thus free radicals scavenging by antioxidants present in the aqueous extracts of cucumbers would contribute to wound healing. Moreover, fatty acids composition analysis revealed that the aqueous extract contained higher content of docosahexaenoic acid (DHA) (**21**) (Figure 4) than the organic extract [130]. It was hypothesized that DHA (**21**) may stimulate pro-inflammatory cytokine production at wound sites, thus helping to control an infection as well as preparing the tissue for further repair by enhancing phagocytosis, stimulating the migration of keratinocytes at wound edges, increasing fibroblast chemotaxis and proliferation, triggering ECM proteins breakdown, as well as regulating the release of other cytokines and growth factors [131]. Besides, eicosapentaenoic acid (EPA) (**22**) and DHA (**21**) (Figure 4), the major fatty acids in sea cucumbers, also intervene in the process of inflammation by stimulation of the production of resolvins (which primarily inhibit IL-1β production) and protectins (which inhibit TNF-α and IL-1β production) through COX-2 and 5-LOX pathways [132]. The study of other species of sea cucumbers also corroborated that their aqueous extracts are more efficient in wound healing than their organic extracts. Furthermore, the anti-inflammatory effect of sea cucumbers in clinical settings was also studied by incorporating sea cucumber extracts into Carbopol^®^ gel base and applied topically to diabetic foot ulcer patients for 12 weeks. The results showed that the levels of TNF-α between the beginning and on weeks 8, 10, and 12 were significantly different [133]. It was suggested that the saponin content in the extracts of sea cucumbers may play a role in preventing the lipopolysaccharide-induced production of TNF-α by nuclear factor-κB (NF-κB), which is a transcription factor that regulates the transcription of many genes involved in the inflammation process [134,135].

Another marine-derived compound with wound-healing capacity is fucoidan (**23**) (Figure 4). Fucoidan (**23**) is a fucose-enriched and sulfated polysaccharide found mainly in the ECM of brown algae. Fucoidan (**23**) is made up of l-fucose, sulfate groups, and one or more small proportions of other sugars [136]. Structurally, fucoidan (**23**) consists of two types of homofucose: those containing repeated (1→3)-l-fucopyranose, and the others consisting of alternating and repeated (1→3)- and (1→4)-l-fucopyranose [137]. This class of polysaccharide has been extensively investigated for its biotechnological potential due to its myriad of pharmacological effects and its non-toxic edible resources [138]. Specifically, low molecular weight fucoidans (LMF), which have a better bioavailability in tissues when compared to high molecular weight fucoidans (HMF), have been found to exhibit beneficial effects such as anti-inflammation and angiogenesis, suggesting their clinical potential for dermal wound healing [108]. In this context, Park et al. have investigated the wound-healing properties of LMF, which was extracted from the marine brown seaweed *Undaria pinnatifida,* in a full-thickness dermal excision rat model in comparison with a commercial product Madecassol Care™, which contains 1% *Centella asiatica*. They have found that the topical application of LMF showed much better effects on wound contraction, in a dose-dependent manner, and faster half-closure time (CT50) when compared to Madecassol Care™, indicating that LMF enhanced the wound healing through its anti-inflammatory activity or promotion of the granulation phase. These results are supported by the fact that fucoidans mediate anti-inflammatory effects via the reduction of neutrophil adhesion and leukocyte recruitment, or the inhibition of pro-inflammatory cytokines release, as previously reported [139]. Additionally, LMF also accelerated angiogenesis and collagen deposition in the increased granular tissue, thus enhancing re-epithelialization. This phenomenon could be explained by the fact that collagen deposition and a formation of tight cross-links among collagen molecules and with other proteins, as well as the proliferation of the various cells in the granular tissue, could enhance wound contraction [140]. On the other hand, MMPs, especially MMP2 and MMP9, are known to be mediators of the collagen matrix during remodeling and wound re-epithelialization [141]. In this context, it was found that treatment with LMF caused an increase in MMP9 on day 7 post-treatment in a dose-dependent manner, suggesting that LMF caused an alteration of the temporal expression of MMP9 to accelerate tissue remodeling in response to the increased secretion of various cytokines or their protection from the proteolytic degradation. Additionally, LMF treatment caused a reduction of the lipid peroxidation (malondialdehyde), while the antioxidant enzymes such as SOD, CAT, and GSH levels were increased. Previous studies have reported that the sulfate contents of fucoidan (**23**) (Figure 4) [142] played an important role in its antioxidant activity, thus the high numbers of sulfate groups in LMF might contribute to its strong antioxidant activity. On the other hand, there is a strong evidence that transforming growth factor beta (TGF-β) and vascular endothelial growth factor (VEGF) are involved in the improvement of wound repair by increasing the fibroblast repopulation and angiogenesis [143], and since LMF treatment also showed a substantial increase in TGF-β and VEGF receptor-2 (VEGFR2) immunoactive cells, this phenomenon could also contribute to its wound-healing capacity.

## 3. Cosmeceuticals from Marine Origin

The increasing awareness of consumers for natural cosmetics has triggered a surge to explore nature’s wealth for biologically active compounds for cosmetic applications [9]. Cosmetics that incorporate marine-based extracts or compounds are increasingly launched by the cosmetics industry as an increasing number of consumers are demanding products from natural sources [6]. A global tendency for products considered healthy, environmentally sustainable, and ecologically friendly obtained has led the cosmetics industry to invest more and more in the research and development (R&D) of new products containing substances or extracts derived from natural resources [144]. Therefore, more and more consumers are looking for cosmetic products with novel bioactive compounds as ingredients obtained from natural resources because of their numerous beneficial effects when compared to their non-natural counterparts. Although plant natural products have been traditionally used (and are still used) as active ingredients for natural cosmetics, marine resources have recently emerged as a rich source of structurally diverse compounds with a myriad of biological properties awaiting to be explored as cosmeceuticals/nutricosmetics. The marine environment represents an extraordinary biodiversity, which is important source of a huge chemical diversity with a great potential for industrial development for pharmaceuticals, cosmeceuticals, nutritional supplements, molecular probes, fine chemicals, and agrochemicals [145]. Moreover, with so many new marine species still to be discovered, more research efforts will be needed to appreciate the vast potentialities that marine environment has to offer [9]. Marine organisms have evolved biochemical and physiological mechanisms that include the production of bioactive compounds necessary for reproduction, communication, and protection against predation, infection, and competition [146]. Not surprisingly, many of these compounds have a great potential as cosmeceuticals or nutricosmetics due to their antioxidant, anti-inflammatory, anti-allergic, antiaging, anti-wrinkle, anti-tyrosinase, MMP inhibitory activities as well as UV protection [147].

Currently, the search for new marine natural products depends on the harvest of specimens whose drawback is their sustainability and replicability. Sustainability issues are associated with large amounts of biomass that are usually required for drug discovery, whereas replicability problems are related with environmental variability and community-level changes to the chemical ecology of the target organisms [148]. Individuals of the same species sampled in different geographical areas or different seasons may not contain the same chemical composition and therefore may not guarantee the supply of the target metabolite [149]. However, recent techniques in aquaculture of marine invertebrates may offer an alternative to overcome these two issues, as animal biomass can be continuously produced using homogenous environmental conditions [150].

### 3.1. Macroalgae-Derived Compounds

In general, marine brown and red algae are commonly used as cosmeceuticals in cosmetic products [151]. Traditionally, macroalgae or seaweeds have been used in the production of phycocolloids such as agar, carrageenan, and alginates. Furthermore, some types of brown and red macroalgae are used in cosmetics due to their vitamins, minerals, amino acids, sugars, and lipids content, in addition to the presence of other biologically active compounds [152,153]. Macroalgae commonly used in cosmetics are *Ulva lactuca, *Ascophyllum* nodosum, Laminaria longicruris, L. saccharina, L. digitata, Alaria esculenta*, *Chondrus crispus*, *Mastocarpus stellatus*, and various species of *Porphyra*. Normally, algae respond to many stress factors, to which they are exposed in natural environments, by the production of a variety of chemical compounds for their defense. Many of these compounds are considered valuable as cosmeceuticals for skincare for protection against UV radiation, oxidative stress and aging, smoothening, moisturizing and whitening, and also as pigments for many cosmetic products [144]. The functional products of macroalgae have been used for decades by the cosmetic industry as emollients, skin conditioning agents, and viscosity controlling ingredients, mainly due to their physicochemical properties. Their bulk products, such as agar and carrageenan, have been used as gelling, thickening, and stabilizer in cosmetic products as well as nutraceuticals [154].

**Figure 5:**
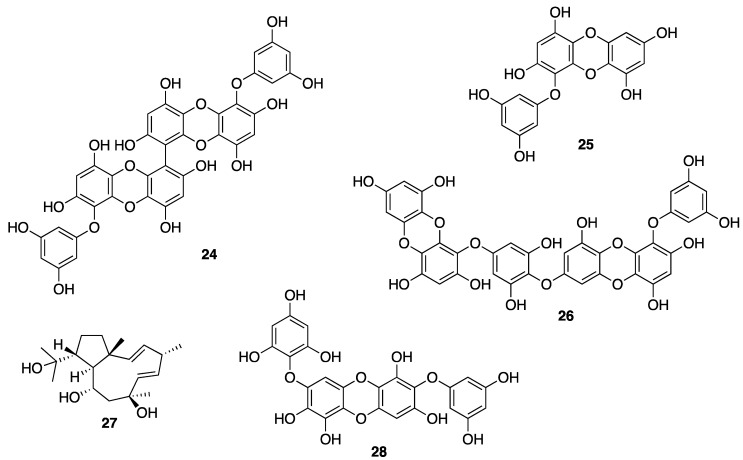
Structures of 6,6´-dieckol (**24**), eckol (**25**), dieckol (**26**), dolabelladienetriol (**27**), and diphlorethohydroxycarmalol (**28**).

Brown algae account for approximately 59% of the total macroalgae cultivated in the world, followed by red algae at 40% and green algae at less than 1%. Macroalgae can be cultivated on seashores in a large scale with a relatively rapid growth rate, and with a possibility to control the production of their bioactive compounds such as proteins, polyphenols, and pigments by manipulating the culture conditions [155]. A lipophilic extract of a brown alga *Alaria esculenta* was effective in the reduction of cutaneous progerin [156], whose over-production is caused by cellular senescence and progressive telomeres damage, which occur naturally [157]. By using a novel gelatin digestion assay to investigate the in vitro inhibitory effects of *Ecklonia cava*-derived phlorotannin on MMP activity, Kim et al. have observed its complete inhibition of bacterial collagenase-1 activity [158]. A sensitive fluorimetric assay revealed that phlorotannin 6,6’-dieckol (**24**) from *E. cava* (Figure 5) can significantly inhibit MMP2 and MMP9 activities through the activation of the NF-κB pathway. Additionally, 7-phloroeckol (**4**) (Figure 1) also exhibited excellent inhibitory effects on pigmentation, which is probably due to its tyrosinase inhibitory activity, and it was proposed as a skin-whitening agent [154,159,160,161]. Eckol (**25**) and dieckol (**26**) (Figure 5), phlorotannins from *E. stolonifera* extract also displayed a strong inhibition of MMP1 expression [162]. Diphlorethohydroxycarmalol (**28**) (Figure 5), a phlorotannin, isolated from a marine brown alga *Ishige okamurae*, exhibited a high potency for the whitening of the skin [163], in addition to protective properties against DNA damage induced by UVB radiation via damaged tail and morphological changes in fibroblasts. These dual biological properties of diphlorethohydroxycarmalol (**28**) make it an interesting cosmeceutical candidate [159]. Phloroglucinol derivatives from brown algae also possess the tyrosinase inhibitory activity due to their ability to chelate copper in this enzyme [164]. In vivo studies have shown that both dietary and topical application of polyphenols from brown algae suppressed COX-2 expression and cell proliferation. These results suggest the role of brown algal polyphenols as potential cancer chemopreventive agents against photocarcinogenesis and other adverse effects of UVB radiation exposure [165]. On the other hand, dolabelladienetriol (**27**) (Figure 5), a dolabellane diterpene isolated from the brown marine alga *Dictyota pfaffii*, was found to downregulate the production of TNF-α and nitric oxide (NO) through an inhibition of NFκB, thus conferring its anti-inflammatory activity [122]. All of these evidences suggest that bioactive compounds derived from seaweeds are promising for skincare [165]. Another important seaweed is *Laminaria japonica*, which is also known as “kombu” in Japan, is used to produce special algae-based active ingredients for protective formulations against UV radiation, since it contains a highly concentrated form of marine minerals and trace elements. As this alga also produces extremely effective moisture binding agents that prevent it from drying out at low tide, its extract could be explored as a potential skin moisturizer as well as to maintain skin firmness [166]. This alga is also a rich source of fucoxanthin (**19**) (Figure 3), which has several beneficial properties for skincare such as antioxidant and anti-tyrosinase activities, antimelanogenesis in melanoma, and anti-UVB-induced skin pigmentation. Moreover, fucoxanthin (**19**) oral tratment significantly suppressed the mRNA expression of melanogenesis-related tyrosinase enzyme, suggesting that this compound negatively regulated the melanogenesis factor at a transcriptional level through the suppression of prostaglandin synthesis and melanogenic stimulant receptors (neurotrophin, PGE2, and melanocyte-stimulating hormone expression) [167]. Another species of *Laminaria* with cosmetic potential is *L. saccharina*, whose extract is rich in proteins, vitamins, minerals, and carbohydrates. The extract of this alga was reported to have anti-inflammatory and healing properties, in addition to regulating sebaceous gland activity [168]. The most common edible brown macroalgae of the *Sargassaceae* family, *Hizikia fusiformis*, was reported to contain the anti-tyrosinase flavonoid glycoside [169]. In vitro studies of the methanol extract of a red marine alga *Corallina pilulifera* (CPM) revealed that it can prevent UV-induced oxidative stress and MMP2 and MMP9 expressions in human dermal fibroblast (HDF) cells. A combination of algal extracts from the red algae *Meristotheca dakarensis* and *Jania* *rubens*, available on the market as dermocea^®^ (Gelyma), was claimed to stimulate keratin, GAGs, and collagens I and III synthesis [170]. All of these studies point to a great potential of marine brown and red algae, in the form of extracts or pure compounds, as valuable marine-derived cosmeceuticals.

Extracts of green algae have also been incorporated in various cosmetic products. The extract of *Codium tomentosum* was claimed to be a good source of glucuronic acid (**38**), and it is used for a distribution of water in the skin as well as to protect the skin from the harmful effects of a dry environment [5]. Extracts of the green alga *Chlamydocapsa* sp., also known as snow alga, are used for topical application to prevent photoaging in the products for skincare and hair protection. Furthermore, it could protect against the loss of the barrier function induced by environmental exposure, decrease TEWL, and avoid wrinkles formation after exposure to UV radiation, cold or dry condition [171].

### 3.2. Marine Invertebrate-Derived Compounds

#### 3.2.1. Marine Sponge-Derived Compounds

**Figure 6:**
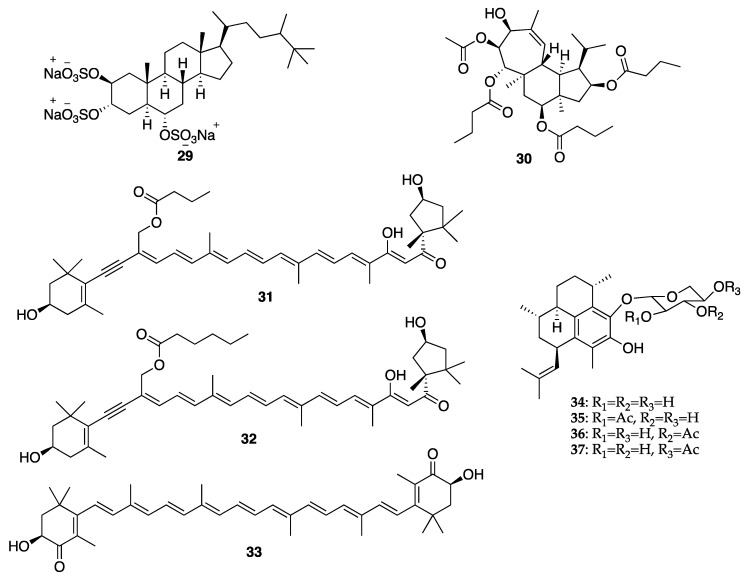
Structures of halistanol trisulphate (**29**), gagunin D (**30**), 19-butanoyloxymytiloxanthin (**31**), 19-hexanoyloxymytiloxanthin (**32**), astaxanthin (**33**), and pseudopterosins A–D (**34**-**37**).

When dealing with secondary metabolites produced by marine invertebrates, especially marine sponges, it is important to consider their relationship with the associated microorganisms and phytoplankton, as some of their isolated bioactive secondary metabolites are suggested to be produced by the functional groups of enzymes originated from the associated microorganisms [172]. These microorganisms can be very important for new pharmaceuticals, cosmeceuticals, and nutraceuticals, because they are renewable resources of different natural products [173,174]. Indeed, marine sponges are considered depositories of marine microbial diversity, which can provide a new avenue in marine biotechnology [175]. This is evidenced by the fact that many sponge-derived metabolites resemble bacterial and fungal natural products or belong to the class of compounds typically produced by these microorganisms [176]. Some reports have confirmed that some compounds, originally isolated from marine sponge extracts, are in fact biosynthesized by sponge-associated microorganisms, since the sponge mesohyl is usually inhabited by microbes, and many natural products isolated from the marine sponges such as antibiotics, antifungal, and antipredator or antifouling compounds seem to be metabolites produced by marine microbes [176]. In the case of bacteria, they provide their hosts with products of their metabolism, thereby granting the sponges an access to bacteria-specific traits such as autotrophy, nitrogen fixation, and nitrification. These bacteria can also process metabolic waste compounds that stabilize the sponge skeleton and provide protection against UV radiation [177,178,179]. In turn, marine sponges also release enzymes to compete for the ground, to delay the growth of bacteria and fungi to present hosting from uninvited guests, and these enzymes can be used as skin-whitening agents in several cosmetic formulations [147]. Although only a few bioactive compounds isolated from marine sponges have been explored by the cosmetics industry so far, there is an increasing number of sponge metabolites with cosmeceutical potentiality. For example, halistanol trisulphate (**29**) (Figure 6), a C-29 steroidal detergent isolated from the Indo-Pacific sponge *Haliclona* sp., was shown to inhibit the maturation of tyrosinase to a form that is associated with melanin synthesis in the pigmented human melanoma cell line, MM418 [180]. Gagunin D (**30**) (Figure 6), a highly oxygenated diterpene of the 10, 13-bis-*epi*-homoverrucosane scaffold, isolated from the marine sponge *Phorbas* sp., was found to exhibit antimelanogenic activity by suppression of the tyrosinase expression and increasing the rate of tyrosinase degradation, in addition to the inhibition of tyrosinase enzymatic activity, in mouse melan-a cells and a reconstructed human skin model [18]. Moreover, gagunin D (**30**) also suppressed the expression of proteins associated with melanosome transfer. Due to its multi-functional properties, gagunin D (**30**) and its analogs can be considered as potential candidates for skin-whitening cosmeceuticals [18]. Marine sponges are also known to produce more than 40 carotenoids [181], most of which are aryl carotenoids such as isorenieratene, renieratene, and renierapurpurin. Since sponges have no biosynthetic machinery to synthesize carotenoids, these pigments are directly accumulated by food intake or through metabolic transformations. Carotenoids play vital roles in marine sponge including the photoprotective and antioxidant functions via light energy dissipation and free radical detoxification due to exposure to excessive solar radiation and harmful UV radiation [182]. Besides aryl carotenoids, the red color pigments mytiloxanthin derivatives, 19-butanoyloxymytiloxanthin (**31**), and 19-hexanoyloxymytiloxanthin (**32**) (Figure 6) were also isolated from the bright orange-colored sponge *Phakellia stellidem* [183]. It is interesting to note that mytiloxanthin, a metabolite of fucoxanthin (**19**) (Figure 3), exhibits almost the same singlet oxygen quenching and lipid peroxidation inhibitory activities as those of astaxanthin (**33**) (Figure 6), but with higher scavenging activity for hydroxyl radical [184]. Consequently, these compounds could have a great value as cosmeceuticals for skincare products.

Marine sponge-derived collagen has also been evaluated for its biocompatibility and regenerative potential [185,186,187]. An in vitro toxicity, antioxidant activity, healing capacity, and photoprotection of trypsin-digested collagen extracts, also called marine collagen hydrolysates (MCHs), from the marine sponge *Chondrosia reniformis* were evaluated [188], due to its particular physicochemical characteristics and dynamic plasticity [189,190]. This study was based on the fact that collagen hydrolysates from various sources demonstrated good biocompatibility, penetration capacity, and protective properties for the skin in different experimental models. It was found that the four MCHs exhibited not only no toxicity and significant antioxidant activity by promoting the elimination of ROS but also a wound-healing capacity by promoting a more accelerated proliferative stage. These data open the way for the application of these MCHs as cosmeceuticals to repair damaged or photoaged skin [188]. Nevertheless, further studies are needed to validate these promising products before they can be launched to the market.

The hexane, methanol, and ethanol extracts of the marine sponge *Acanthella cavernosa* were evaluated for their antibacterial and antibiofilm activities against *P. acnes*, as well as their antioxidant activity; however, only the ethanol extract exhibited the in vitro antibacterial and antibiofilm activities against this bacterium. Therefore, this marine sponge has a potential to be applied as a natural marine-derived cosmeceutical for acne prevention [191].

#### 3.2.2. Coral-Derived Compounds

Coral powder is used as a sustainable material in numerous cosmetic products due to its physical, chemical, and textural characteristics as well as its mineral content [147]. Chemically, it is composed mainly of calcium carbonate but may contain about 74 other minerals, except heavy metals. Coral powder is used for a topical application to provide minerals for the skin, to protect against UV radiation and also as antioxidant, antiaging, antiacne, skin softening, as well as for the preparation of lipsticks and deodorants [147]. Although only few coral secondary metabolites have found their use as cosmeceuticals, the diterpene glycosides pseudopterosins A–D (**34**–**37**) (Figure 6), isolated from the Caribbean Gorgonian coral *Pseudopterogorgia elisabethae*, are the most notable marine natural products in the cosmetic industry [192]. These compounds possess a variety of biological activities ranging from anti-inflammatory and analgesic [193,194,195], antibacterial [196], antiacne [197] to wound healing [198,199]. These compounds are the first commercially licensed natural products for use as an additive in Estée Lauder skincare and anti-wrinkle cosmetic product under the brand name Resilience^®^ [200]. However, the most studied member of this class of compounds for their anti-inflammatory activity was pseudopterosin A (**34**) (Figure 6), which inhibited phagosome formation and triggered intracellular calcium release by a mechanism that involved its binding to the G protein-coupled receptor [201]. Other pseudopterosins with exceptional anti-inflammatory activity have been also identified and are suggested to inhibit the synthesis of leukotrienes and degranulation of human neutrophils [122].

#### 3.2.3. Sea Cucumber-Derived Compounds

Sea cucumbers are also rich in bioactive compounds such as saponins, chondroitin sulfate, collagen, vitamins, amino acids, phenols, triterpene glycosides, carotenoids, bioactive peptides, minerals, fatty acids, and gelatin. Among the health benefits of sea cucumbers are wound healing, neuroprotective, antitumor, anticoagulant, antimicrobial, and antioxidant properties [202]. Sea cucumber extracts are rich in vitamins A, B1 (thiamine), B2 (riboflavin), B3 (niacin), and minerals (calcium, magnesium, iron, zinc, selenium, germanium, strontium, copper, manganese) that can be used as cosmeceuticals or nutricosmetics. The vitamins and minerals in sea cucumber extracts are easy to be absorbed and provide moisture while stimulating the renovation of damaged skin cells [6]. The investigation conducted on the Red Sea cucumber (*Stichopus japonicus*) extract showed a remarkable inhibition of melanogenesis in melanoma and inhibited the expression of tyrosinase and tyrosinase-related proteins (TYRP-1 and TYRP-2). Yoon et al. demonstrated that the ethyl acetate fraction of the *S. japonicus* extract inhibited melanogenesis in murine melanoma cells, decreasing the protein level of the melanocyte-specific isoform of the tyrosinase-related genes [203]. Evaluation of the skin-whitening effects of the extracts of *Sanguisorba officinalis* and *Stichopus japonicus* showed that the extract of *S. japonicus* exhibited 61.78% inhibition of tyrosinase activity, while the mixture of both extracts showed 59.14% inhibition. Interestingly, the mixture of both extracts displayed a notable inhibition of melanogenesis in the clone M-3 cell melanocyte [204]. A glycoprotein fraction of boiled *S. japonicus* has been shown to enhance tyrosinase inhibitory activity by 50% [205]. The tyrosinase inhibition exhibited by the bioactive extracts of sea cucumber species makes them promising skin-whitening cosmeceuticals with numerous advantages such as low cytotoxicity, high safety, and wide acceptance.

**Figure 7:**
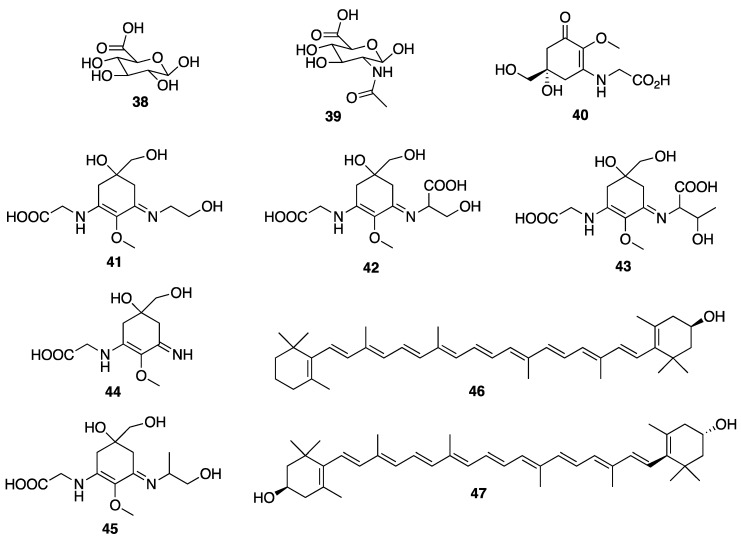
Structures of β-d-glucuronic acid (**38**), *N*-acetyl-β-d-glucosamine (**39**), mycosporine-glycine (**40**), asterina-330 (**41**), shinorine (**42**), porphyra-334 (**43**), palythine (**44**), palythinol (**45**), β-cryptoxanthin (**46**), and xanthophyll (**47**)**.**

Another important aspect of sea cucumbers is their considerable amount of novel sulfated polysaccharides, which have great potential for the development of cosmeceuticals and pharmaceuticals. The sulfated polysaccharides isolated from the body wall of sea cucumbers, named fucosylated chondroitin sulfates (FuCS), are structurally different from sulfated polysaccharides isolated from other invertebrates, vertebrates, and algae [206]. The high amount of these sulfated glycans can be separated into three fractions: the first fraction has a high amount of fucose, the second contains primarily fucoidan (**23**) (Figure 4), and the third has a high proportion of glucuronic acid (**38**) [206]. FuCS were isolated from several sea cucumber species, including *Ludwigothurea grisea*, *Pearsonothuria graeffei*, *Holothuria vagabunda*, *H. edulis, H. nobilis*, *Stichopus tremulus*, *S. japonicus*, *Isostichopus badionotus*, *Thelenata ananas*, *Apostichopus japonicas*, *Acaudina molpadioidea*, and *Athyonidium chilensis*. Structurally, FuCS are composed of repetitive units of β-d-glucuronic acid (**38**) and *N*-acetyl-β-d-glucosamine (**39**) (Figure 7) [207,208]. It has been reported that sea cucumber fucoidan (Figure 4) exhibits numerous biological activities [209,210]. For example, fucoidan (**23**) from *Thelenota ananas* was shown to possess a significant superoxide radical scavenging activity, which is improved with the increasing sulfate content. Moreover, additional 2-*O*-sulphation in a specific residue increases the radical scavenging effect, suggesting that the antioxidant activity of fucoidan (**23**) derived from *T. ananas* depends on the sulfation pattern and not simply on the sulfate content [211]. Moreover, the sulfate content and structural feature of fucoidan (**23**) (Figure 4) have a profound relationship with its biological properties. Fucoidan (**23**) isolated from *S. japonicus*, *I. badionotus*, and *L. grisea* showed interesting biological activities that can be exploited as cosmeceuticals [209]. As fucoidan (**23**) could increase the MMP1 activity in human skin, it can be used as an antiaging agent to prevent wrinkle formation and skin photoaging for cosmetic products [209,210].

Sea cucumbers have been reported to have high amounts of collagen and mucopolysaccharides that are relatively safe when compared with animal collagen [42,57]. The total protein of the body wall of sea cucumbers contains approximately 70% of insoluble collagen fibers, which can be converted into gelatin after hydrolysis. Collagen fibers are hardly soluble due to the intermolecular cross-links formed by non-helical telopeptides of adjacent collagen molecules, whereas gelatin is a soluble protein obtained by the partial hydrolysis of collagen [212,213]. Studies on sea cucumber collagen have been mainly focused on the functions of its hydrolytic bioactive peptides, including damaged tissue repairing, antitumor, antioxidant, and angiotensin-converting enzyme inhibitory activities. Due to their antioxidant property, collagen fibers have been used in skincare products [214]. Another group of constituents of sea cucumbers is saponins [215]. These compounds play an important role in chemical defense and possess a wide spectrum of pharmacological activity. The majority of sea cucumber saponins are usually triterpene glycosides of the holostane type [215]. Some saponins can decrease dandruff and alleviate psoriasis when applied topically, in addition to decreasing hyperpigmentation, rosacea, strengthening blood vessels, and improving water penetration. Since a majority of the research studies on pharmacological activity has been conducted on plant saponins, more in-depth research on saponins from sea cucumbers is necessary to verify if they have the same beneficial effects as their plant counterparts.

The ecological concept also plays a fundamental role in searching for bioactive compounds useful for cosmeceuticals and nutricosmetics. Example of this is an observation that MAAs have a protective role in many marine organisms such as the holothuroids, especially the black sea cucumber *Holothuria atra* [216], where they occur predominantly in its epidermal tissues. The epidermal tissue of *H. atra* contains varied amounts of several MAAs such as mycosporine-glycine (**40**), asterina-330 (**41**), shinorine (**42**), porphyra-334 (**43**), palythine (**44**), and palythinol (**45**) (Figure 7), whereas the ripe ovaries and brooded juveniles of *Cucumaria ferrari* contain moderate amounts of mycosporine-gly (**40**), shinorine (**42**), porphyra-334 (**43**), and palythine (**44**) [217]. Sunscreen formulations containing liposomes of porphyra-334 (**43**), obtained from sea cucumbers, were found to diminish skin lipid oxidation and skin aging parameters such as low elasticity, wrinkle depth, and roughness. Upon irradiation, reactive intermediates were not produced by porphyra-334 (**43**), suggesting that this compound transformed absorbed UV radiation into harmless thermal energy [218]. Extracts of some sea cucumber species, especially *S. hermanni*, *H. fuscogilva*, *A. mauritiana*, *A. crassa*, *B. vitiensis*, *B. tenuissima*, *P. graeffei*, *B. cousteaui*, *H. atra*, *H. leucospilota*, and *H. nobilis* exhibited potent antibacterial activity [219,220]. The report showed that β-cryptoxanthin (**46**), xanthophyll (**47**) (Figure 7), and β-carotene (**7**) (Figure 2), isolated from the Egyptian sea cucumber *H. scabra*, exhibited strong antibacterial activity against *S. aureus* (ATCC 6538) [219]. This finding can be important for the use of carotenoids-containing sea cucumber extracts to prevent the microbial contamination in cosmetics that can cause deterioration of the products and pose a serious risk to the consumers [221].

As mentioned earlier, some bioactive metabolites of sea cucumbers can induce tissue repair and enhance the wound-healing process. It has been reported that GAGs of the integumentary tissue of *S. vastus* and *S. hermanni* exerted wound-healing properties in rats [123,222]. Masre et al. reported that part of the sea cucumber tegument had the highest total *O*-sulfated GAG content, followed by internal organs and coelomic fluid [222].

### 3.3. Marine Microorganisms-Derived Compounds

Marine microorganisms, including fungi, fungi-like protists, and bacteria have attracted great attention as potential lead compound producers [223,224]. Despite a relatively small number of the species of these organisms being studied so far, thousands of compounds have been isolated and identified, among which only a small percentage has been investigated for their potential as commercially useful products [225]. Anyhow, the popularity of marine ingredients has caused concerns that large-scale sourcing or non-sustainable production methods could disrupt marine ecosystems that are already under strain. Since many marine microorganisms are cultivable and can be cultured in fermenters, they present a great advantage as a sustainable resources to produce high-valued compounds [226].

#### 3.3.1. Microalgae-Derived Compounds

The diversity of microalgae makes them a rich source of bioactive compounds with potential applications as nutraceuticals and cosmeceuticals. Microalgae also constitute major food products, mainly for animal feed due to their fatty acids, tocopherols, sterols, proteins, carbohydrates, vitamins, minerals, antioxidants, and pigments (e.g., chlorophyll and carotenoids) contents [227]. Microalgae, including *Chlorella*, *Spirulina*, *Dunaliella,* and *Odontella* species have also been used as ingredients in cosmetics [33]. In terms of cosmeceuticals, microalgae are of great interest as some of them synthesize substances that absorb UV radiation, which can prevent dermal ECM deterioration, wrinkles, laxity, coarseness, and mottled pigmentation of the skin. For example, the cyanobacterial sunscreen pigment scytonemin (**6**) (Figure 2) absorbs UVA/UVB radiation more efficiently than a commercial formulation [228]. Scytonemin (**6**) is produced by several cyanobacteria such as *Nostoc* sp., *Calothrix crustacean,* or *Chlorogloeopsis* sp. [229]. Another UV protection pigment is β-carotene (**7**) (Figure 2), the main carotenoid produced by the halotolerant microalga *Dunaliella salina,* which can produce more than 10% of β-carotene (**7**) of its dry weight [230]. Another well-known carotenoid produced by microalgae is astaxanthin (**33**) (Figure 6). This compound has been extensively studied for its beneficial effects on skin health as well as for its photoprotective effects against UV radiation [231]. As astaxanthin (**33**) can improve skin health by influencing the various stages of the oxidative damage cascade, in addition to suppressing various inflammatory mediators [232], it was therefore considered a strong antioxidant and an excellent anti-inflammatory agent. Moreover, it also exhibits immunomodulatory and DNA repair properties, which further supports its use to maintain skin health and to prevent skin damage [231]. *Haematococcus pluvialis* accumulates large amounts of astaxanthin (**33**), and it is considered as the main natural source for human consumption. Due to a huge demand of astaxanthin (**33**) as cosmeceutical/nutricosmetic, a sustainable production of astaxanthin (**33**) by *H. pluvialis* has already reached an industrial scale [233].

**Figure 8:**
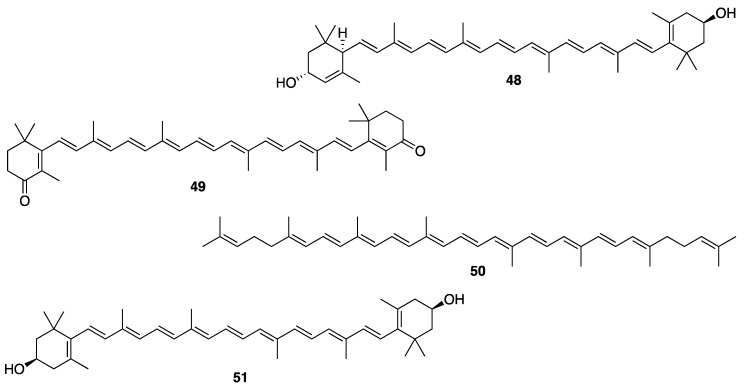
Structures of lutein (**48**), canthaxanthin (**49**), lycopene (**50**), and zeaxanthin (**51**).

Other carotenoids such as lutein (**48**), canthaxanthin (**49**), lycopene (**50**), and zeaxanthin (**51**) (Figure 8) have also gained some importance in the health and cosmetics sectors [38]. Lutein (**48**) has been shown to protect the epidermal and dermal layers of the skin against UV-induced oxidative damage, especially in combination with other antioxidant and immunoprotective substances [234]. Microalgae also possess a moisturizing property that can improve and maintain the barrier function of the skin, hair, etc., keeping them in a healthy appearance. For example, some proteins and their hydrolysates from *Spirulina* sp. confer a moisturizing property in hair products providing the retention of water, and they are recommended for atopic dermatitis or other dry skin conditions [144,235]. Oils obtained from the dried material of intact cells of some microalgae, especially form the genus *Chlorella*, have softening and smoothening properties for skin and hair [236,237]. Another emerging marine cosmeceutical for topical application for skincare cosmetic is alguronic acid. Alguronic acid is not a pure compound but a trade name created for an undetermined mixture of polysaccharides produced by microalgae by “Solazyme” (currently TerraVia Holdings, Inc.). In 2011, the acid was introduced to the market as an active ingredient in a commercial product called Algenist antiaging skincare formula [79].

The pure extract of the marine microalga *Nannochloropsis oculata* contains zeaxanthin (**51**) (Figure 8) and PUFA-containing lipids which are rich in EPA (**22**) (Figure 4) [238,239]. The extract of this microalga, cultivated in special photobioreactors where they are optimally exposed to light and CO_2_, was licensed for a natural skincare ingredient PEPHA^®^-TIGHT [240] for antiaging formulation taking advantage of a combination of the anti-tyrosinase and antioxidant properties of zeaxanthin (**51**) and a moisturizing effect of EPA (**22**).

The extract of the marine diatom *Phaeodactylum tricornutum*, rich in fucoxanthin (**19**) (Figure 3) [241] and ω-3 PUFAs such as EPA (**22**) and DHA (**21**) (Figure 4) [242], was found to promote proteasome activity in skin cells, particularly keratinocytes, fibroblasts, or melanocytes. This extract can protect the skin from the adverse effects of UV radiation exposure in addition to improving the elasticity and firmness of the skin. It may delay the appearance of wrinkles and/or reduce their depth [243]. The extract of *P. tricornutum* is used as an ingredient in two antiaging and revitalizing creams for skincare, i.e., Depollutine^®^ and Megassane^®^ [244].

Extracts of the diatoms *Thalassiosira* sp. and *Chaetoceros* sp. and of the microalgae *Chlorococcum* sp. and *Monodus* sp., which contain fucoxanthin (**19**) and other carotenoid pigments [245,246,247] and ω-3 PUFAs such as DHA (**21**) and EPA (**22**) [248,249], are proposed for formulations to prevent hair loss, since they could modulate melanogenesis in hair and skin, improving and stimulating keratinocyte differentiation, melanocyte proliferation, and the growth of hair and hair follicles [250].

#### 3.3.2. Marine Bacteria-Derived Compounds

Marine bacteria are abundant on the surface of the sea but decrease in number with increasing depth, and most of them are associated with organic particles or zooplanktons as their substrate. Marine bacteria are prolific producers of secondary metabolites for their own defense against other microorganisms as they thrive in harsh oceanic climates, and these secondary metabolites can serve as a good source of bioactive compounds [223]. A large number of bacterial bioactive secondary metabolites have high commercial value and have found their place in pharmaceutical and cosmetics industries [5]. Indeed, many compounds derived from marine bacteria such as alkaloids, peptides, proteins, lipids, mycosporines, and MAAs, glycosides, and isoprenoids exhibit photoprotective, antiaging, antimicrobial, antioxidant, and moisturizing activities [251].

**Figure 9:**
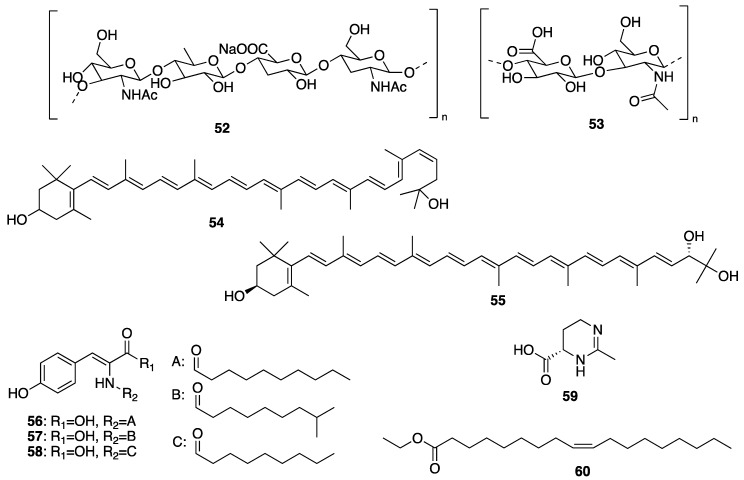
Structures of exopolysaccharide (**52**), hyaluronic acid (**53**), saproxanthin (**54**)**,** myxol (**55**), thalassotalic acids A (**56**), B (**57**), and C (**58**), ectoine (**59**), and ethyl oleate (**60**).

Among the bioactive compounds with antiaging activity of the marine origin, polysaccharides (PSs) are one of the most exploited cosmeceutical products [252], and bacteria are the most favorable organisms for the production of higher PSs [253]. Deepsane, an exopolysaccharide derived from the marine bacterium *Alteromonas macleodii*, is commercially available under the name Abyssine^®^ [1] for soothing and reducing the irritation of sensitive skin against chemical, mechanical, and UVB aggression [254,255]. A mixture of PSs from *Pseudoalteromonas* sp., isolated from Antarctic waters, is incorporated in the formulation of antiaging products. This mixture, obtained through fermentation, is able to enhance the synthesis of collagen I, contributing to the amelioration of skin structural properties [254]. A deep-sea hydrothermal vent marine bacterium, *Vibrio diabolicus*, produces an exopolysaccharide HE 800 (**52**) (Figure 9) that is structurally analogous to hyaluronic acid (**53**) (Figure 9), with unique functions that stimulate collagen structuring [256].

Two rare carotenoids with relevant antioxidant activity, saproxanthin (**54**) and myxol (**55**) (Figure 9), were isolated from new strains of marine bacteria belonging to the family *Flavobacteriaceae.* The addition of saproxanthin (**54**) or myxol (**55**) to cosmetics might help reinforcing biological membranes, decreasing permeability to oxygen and enhancing protection against oxidation. Interestingly, the antioxidant activity of saproxanthin (**54**) and myxol (**55**) is even greater than that of zeaxanthin (**51**) (Figure 8) and β-carotene (**7**) (Figure 2) [257]. Astaxanthin (**33**) (Figure 6) is also produced by some marine-derived bacteria such as *Paracoccus* sp. [258] and *Agrobacterium* sp. [259].

Methylene chloride, produced by new species of the marine bacteria (*Pseudomonas* sp.), can reduce the pigmentation of human melanocytes and cultured skin cells by inhibiting the expression of tyrosinase [260]. The *N*-acyldehydrotyrosine analogues, thalassotalic acids A (**56**), B (**57**), and C (**58**) (Figure 9), isolated from the marine bacterium *Thalassotalea* sp., which was obtained from a bivalve, exhibited the anti-tyrosinase activity. Interestingly, the anti-tyrosinase activity of thalassotalic acid A (**52**) is comparable to that of the commercially used control compound, arbutin (**1**) (Figure 1). The authors suggested that the presence of a carboxylic acid and a linear aliphatic chain contribute to an increase of the enzymatic inhibition within this structural class of compounds [261]. Another bacteria-derived compound is ectoine or 1,4,5,6-tetrahydro-2-methyl-4-pyrimidinecarboxylic acid (**59**) (Figure 9), which is an osmo-protectant produced by several bacterial species in response to osmotic stress [262]. Ectoine (**59**) was first isolated from *Ectothiorhodospira halochloris* but has been also isolated from other halophilic bacteria such as α- and γ-proteobacteria and some Actinobacteridae under high salt concentrations [263]. This compound improves the hydration of the cell surface by increasing intermolecular spacing and boosts the mobility of the lipid head groups in the cell membrane [262], and it is well tolerated by humans [264,265,266]. Thus, ectoine (**59**) is an effective long-term moisturizer that prevents dehydration of the epidermis [262,267]. It also reduces skin inflammation and is currently being investigated for the treatment of moderate atopic dermatitis [264].

Fatty acid esters are common ingredients in cosmetic formulations as natural emollient and emulsifiers [79]. Although many fatty acids esters currently used in cosmetics are obtained from higher plants, some bacteria can also produce unique fatty acid esters. Thus, ethyl oleate (**60**) (Figure 9), which is widely used in many cosmetic products as emollient and perfuming, was also obtained from actinomycetes *Nocardiopsis dassonvillei*, which is a symbiont of the marine sponge *Dendrilla nigra*. This compound also displayed anti-inflammatory activity [268]. Therefore, ethyl oleate (**60**) could be a potential multifunctional cosmeceutical for skincare products that can be produced in a sustainable way.

Many marine sponge-derived *Actinomycetes* sp. and *Streptomyces* sp. have been also investigated as a renewable sources of carotenoids for biotechnological products such as food- and cosmetic-grade natural pigments [269,270].

#### 3.3.3. Marine Fungi-Derived Compounds

**Figure 10:**
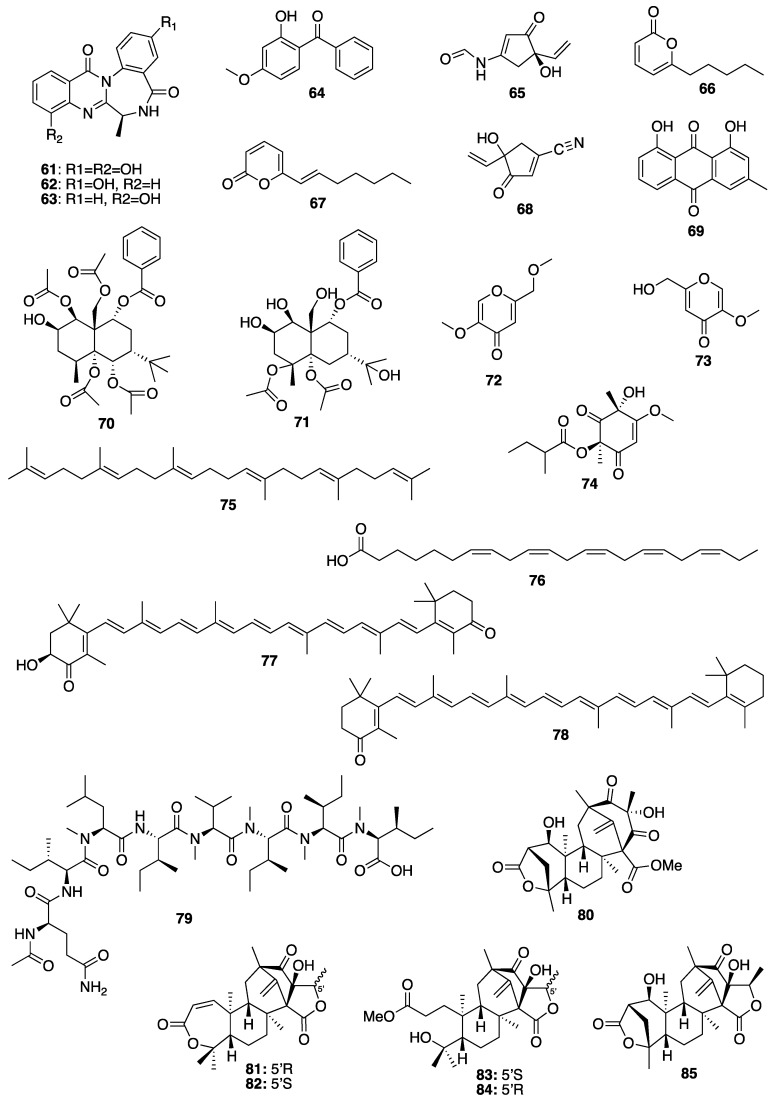
Structures of circumdatins I (**61**), C (**62**) and G (**63**), oxybenzone (**64**), myrothenone A (**65**), 6-n-pentyl-α-pyrone (**66**), 6-[(E)-hept-1-enyl]-α-pyrone (**67**), homothallin II (**68**), chrysophanol (**69**), 1β, 5α, 6α, 14-tetraacetoxy-9α-benzoyloxy-7β H-eudesman-2β, 11-diol (**70**), 4α, 5α-diacetoxy-9α-benzoyloxy- 7βH-eudesman-1β, 2β, 11, 14-tetraol (**71**), kojic acid dimethyl ether (**72**), kojic acid monomethyl ether (**73**), phomaligol A (**74**), squalene (**75**), docosapentaenoic (**76**), phoenicoxanthin (**77**), echinenone (**78**), RHM1 (**79**) and brasilianoids A–F (**80**–**85**).

Several marine-derived fungi produce secondary metabolites with cosmeceutical potential. For example, *Phaeotheca triangularis*, *Trimmatostroma salinum*, *Hortaea werneckii*, *Aureobasidium pullulans,* and *Cryptococcus liquefaciens* are known to produce MAAs [271]. The benzodiazepine alkaloids, circumdatins I (**61**), C (**62**), and G (**63**) (Figure 10), isolated from the culture of the marine sponge-associated fungus *Exophiala* sp. (Family: Herpotrichiellaceae) displayed more potent UVA protecting activity than the positive control oxybenzone (**64**) (Figure 10), which is currently used in sunscreen formulations [272]. Myrothenone A (**65**) and 6-*n*-pentyl-α-pyrone (**66**) (Figure 10), isolated from the culture of the algicolous fungus *Myrothecium* sp. which was obtained from the marine green alga *Enteromorpha compressa*, exhibited stronger anti-tyrosinase activity (IC_50_ = 6.6 and 0.8 µM, respectively) than kojic acid (**3**, IC_50_ = 7.7µM) [273]. The culture of the fungus *Botrytis* sp., isolated from the surface of the red seaweed *Hyalosiphonia caespitosa*, furnished 6-[(*E*)-hept-1-enyl]-α-pyrone (**67**) (Figure 10), which also exhibited more potent anti-tyrosinase activity than kojic acid (**3**) [274]. Culture of the marine sediment-derived *Trichoderma viridae* H1-7 produced homothallin II (**68**) (Figure 10), which is a competitive inhibitor of the mushroom tyrosinase. This compounds appeared to inhibit the enzyme by binding to a copper active site of the enzyme [275]. Although only a few marine fungal metabolites have made their way to the cosmetics world, a patent of a skin-whitening agent, chrysophanol (**69**) (Figure 10), extracted from the algicolous fungus *Microsporum* sp. (MFS-YL), was filed in the USA (U.S. patent 20140056834A1) [10]. By adding the abiotic stressor, CuCl_2_, to the culture of the marine-derived fungus *Pestalotiopsis* sp. Z233, isolated from the marine alga *Sargassum horneri*, two previously unreported sesquiterpenes, 1β, 5α, 6α, 14-tetraacetoxy-9α-benzoyloxy- 7β H-eudesman-2β, 11-diol (**70**) and 4α, 5α-diacetoxy-9α-benzoyloxy-7βH-eudesman-1β, 2β, 11, 14-tetraol (**71**) were obtained (Figure 10). Compounds **70** and **71** exhibited inhibitory activity against the mushroom tyrosinase with IC_50_ values of 14.8 µM and 22.3 µM, which are comparable to that of kojic acid (**3**, IC_50_ = 21.2 µM) [276]. A culture broth of the marine fungus *Alternaria* sp., isolated from the surface of a marine green alga *Ulva pertusa*, produced two kojic acid derivatives, i.e., kojic acid dimethyl ether (**72**) and kojic acid monomethyl ether (**73**), together with phomaligol A (**74**) (Figure 10); however, only kojic acid displayed anti-tyrosinase activity [277].

Squalene (**75**) (Figure 10), which was originally obtained from shark liver oil, can be also obtained from microorganisms such as the protista *Thraustochytriales.* As a common lipid produced by sebaceous glands, squalene (**75**) plays an important role in topical skin lubrication and cellular structure and protection. Thus, squalene (**75**) is used in cosmetics to keep skin moisturized. Moisturizing creams containing squalene (**75**) are non-toxic, non-irritating, and non-sensitizing, while providing antistatic and emollient properties [278]. On the other hand, fatty acids are known not only for their use as dietary supplements but also for their broad spectrum cosmeceuticals due to their role in soft tissue repair and skin nourishment through the stimulation of collagen production as well as anti-inflammatory and wound-healing properties [279]. *Thraustochytrids,* or fungi-like protists, have been explored for the industrial production of PUFAs such as DHA (**21**), EPA (**22**) (Figure 4), and docosapentaenoic (DPA) (**76**) (Figure 10) due to their high production per unit of biomass [280,281]. In particular, the species belonging to *Schizochytrium*, *Aurantiochytrium*, and *Ulkenia*, from the Thraustochytriaceae family, are the main producers of DHA (**21**) [282]. DHA-rich oils from *Thraustochytrids* are currently on the market as nutraceuticals; however, they also have a great potential as cosmeticeutical and nutricosmetic [283]. *Thraustochytrids* such as *Thraustochytriidae* sp. ONC-T18, CHN-1, *Ulkenia* sp. AS4-A1 and *Aurantiochytrium* sp. KH105) also produce carotenoids, including β-carotene (**7**) (Figure 2), astaxanthin (**33**) (Figure 6), canthaxanthin (**49**), and zeaxanthin (**51**) (Figure 8), phoenicoxanthin (**77**), and echinenone (**78**) (Figure 10), which can be used as photoprotective and antioxidant ingredients in different cosmetic formulations [283].

A highly *N*-methylated linear octapeptide RHM1 (**79**) (Figure 10), isolated from the culture of a marine-derived *Acremonium* sp., which was obtained from an unidentified marine sponge from Papua New Guinea, exhibited antibacterial activity against *S. epidermidis,* which is a causative agent of acne [105]. Another class of fungal metabolites with a myriad of biological activities is meroterpenoids. Recently, Zhang et al. [284], in their search for bioactive secondary metabolites from the culture of a marine sponge-associated fungus *Penicillium brasilianum* WZXY-m122-9, have isolated a series of meroterpenoids, which they have named brasilianoids A–F (**80**–**85**) (Figure 10). Interestingly, only **80** showed significant stimulation of the expression of filaggrin, which is an essential natural moisturizing factor that maintains the ability to regulate the skin’s moisture barrier [285], and of caspase-14, which is responsible for controlling TEWL and for sensitivity to UVB damage [286]. Thus, this compound is the first example of a natural product that can be used to promote the protection of UVB-induced cell damage, suggesting that it can have a great potential as cosmeceutical for skincare and for the treatment of dermatological diseases [284].

#### 3.3.4. Yeasts-Derived Compounds

Several genera of yeasts, namely *Rhodotorula*, *Phaffia*, and *Xanthophyllomyces* are known to produce astaxanthin (**33**) (Figure 6) [11]. Although yeasts produce lower amounts of astaxanthin (**33**) when compared to other organisms such as algae, they have several advantages over other organisms, since they have higher growth rates, easier cultivation conditions, and can be genetically modified or by gene target uncovering to increase carotenoid production rates [287,288,289].

## 4. Future Perspectives and Conclusions

As the baby-boomer generation is entering their advanced age, the desire to look younger and healthier has become the global priority. The influence of the social media to inform the population and an effective dissemination of scientific research have raised the awareness of the risk of using many chemicals in drugs and cosmetics as well as health benefits of compounds obtained from natural resources. Thus, this millennial is marked by environmentally friendly processes and the use of natural substances. For an alternative to the “green technology”, marine or “blue biotechnology” is gaining its turf by providing a myriad of natural products that cannot be found in terrestrial environments and with unprecedented biological and pharmacological properties. Although the pharmaceutical sector has been a pioneer in exploiting the treasures from the oceans, the cosmetic and nutraceutical sectors have now paying more attention to the marine environments.

In spite of some products of a marine origin have already appeared on the market, the number of these products is still very timid when compared to the vastness of the sea and the future discoveries that lie ahead. For example, until 2012, only three types of compounds from marine algae were commercially exploited, i.e., alginates, agar, and carrageenan. This demonstrates that there are still many marine compounds, especially small molecules, which can be exploited as cosmeceuticals and nutricosmetics. However, more efforts for the isolation and characterization of the products to discover the pharmacophores, molecular modifications, evaluation of their pharmacological properties and safety aspect, improvement of the quality of products, and above all, more investment in R&D is much needed. It is interesting to mention also that these marine resources are still poorly exploited due to some inherent limitations. First and foremost, the quantity of the compounds isolated from biological materials, which are normally collected from the marine environments, is very small and thus makes it difficult for further bioassays and development. Secondly, the variations of their production that are influenced by environmental changes to which marine organisms are exposed. So, there is a need to find a sustainable way such as the farming of marine organisms with optimal conditions to harvest bioactive metabolites to be used as active ingredients, excipients, and additives. In this aspect, microbial biotechnology can be considered a promising avenue for obtaining good quantity of high-valued compounds as cosmeceuticals and nutricosmetics.
